# Long-term Follow-up of Psychiatric Disorders in Children and Adolescents Conceived by Assisted Reproductive Techniques in Sweden

**DOI:** 10.1001/jamapsychiatry.2021.3647

**Published:** 2021-12-15

**Authors:** Chen Wang, Anna L. V. Johansson, Kenny A. Rodriguez-Wallberg, Mikael Landén, Catarina Almqvist, Sonia Hernández-Díaz, Anna S. Oberg

**Affiliations:** 1 Department of Medical Epidemiology and Biostatistics, Karolinska Institutet, Stockholm, Sweden; 2 Department of Oncology-Pathology, Karolinska Institutet, Stockholm, Sweden; 3 Department of Reproductive Medicine, Division of Gynecology and Reproduction, Karolinska University Hospital, Stockholm, Sweden; 4 Department of Psychiatry and Neurochemistry, University of Gothenburg, Gothenburg, Sweden; 5 Astrid Lindgren Children’s Hospital, Karolinska University Hospital, Stockholm, Sweden; 6 Department of Epidemiology, Harvard T. H. Chan School of Public Health, Boston, Massachusetts

## Abstract

**Importance:**

Individuals conceived with assisted reproductive techniques (ARTs) could be at elevated risk of psychiatric disorders owing to potential adverse effects of the procedures themselves, or because such traits or their risk factors may be more common in couples with infertility.

**Objective:**

To investigate the risk of psychiatric disorders in adolescents and young adults conceived with ARTs and to evaluate the role of treatment-related parental characteristics.

**Design, Setting, and Participants:**

This prospective follow-up of a nationwide birth cohort used linkage of Swedish population registers with coverage through 2018. All children born in Sweden from January 1, 1994, to December 31, 2006, were included in the analysis. Follow-up was completed on December 31, 2018, when participants were 12 to 25 years of age, and data was analyzed from March 17, 2020, to September 10, 2021.

**Exposures:**

In vitro fertilization with or without intracytoplasmic sperm injection and transfer of fresh or frozen-thawed embryos.

**Main Outcomes and Measures:**

Clinical diagnoses of mood disorder, including major depression, anxiety, obsessive-compulsive disorder (OCD), or suicidal behavior, were identified from hospital records and outpatient specialist care. Suicide was additionally identified from death certificates. Antidepressant use was identified from dispensations of prescribed medications.

**Results:**

A total of 1 221 812 children (48.6% female, 51.4% male) born between 1994 and 2006 were followed up to a median age of 18 (IQR, 15-21) years. Among these participants, 31 565 (2.6%) were conceived with ART. Compared with all others, adolescents conceived with ART had an elevated risk of OCD (hazard ratio [HR], 1.35 [95% CI, 1.20-1.51]), but the association was attenuated and no longer statistically significant after adjustment for parental characteristics (adjusted HR [aHR], 1.10 [95% CI, 0.98-1.24]) and was no longer present when restricted to individuals born to couples with known infertility (aHR, 1.02 [95% CI, 0.89-1.17]). Adolescents conceived with ARTs were not at elevated risk of depression or suicidal behavior compared with other adolescents (irrespective of parental infertility). Type of fertilization (standard in vitro fertilization or intracytoplasmic sperm injection) had no association with outcomes. Compared with non–ART-conceived children of couples with infertility, fresh, but not frozen, embryo transfer was associated with a lower risk of mood disorders (aHR, 0.90 [95% CI, 0.83-0.97]), making frozen embryo transfer appear less advantageous when directly contrasted with fresh embryo transfer.

**Conclusions and Relevance:**

These findings suggest that adolescents conceived with ARTs around the millennium are not at risk of poor psychiatric health compared with the general population, except for an elevated risk of OCD that may be explained by differences in parental characteristics.

## Key Points

**Question:**

Are children conceived with assisted reproductive techniques at risk of poor psychiatric health owing to adverse effects of the treatment or parental background factors?

**Findings:**

In this long-term follow-up of a Swedish birth cohort of 1 221 812 children, those conceived with assisted reproductive techniques had an elevated risk of obsessive-compulsive disorder (OCD) but not of any other types of anxiety, depression, or suicide compared with all other children. The risk of OCD was no longer present in comparison with children born to couples with infertility.

**Meaning:**

These findings suggest that adolescents conceived with ART are not at risk of poor psychiatric health compared with the general population, except for an elevated risk of OCD that may be explained by differences in parental characteristics.

## Teaser

This cohort study quantifies the occurrence of psychiatric disorders, use of antidepressants, and suicidal behavior among children and adolescents in Sweden who were conceived by assisted reproductive techniques and assesses whether outcomes are associated with parental characteristics.

## Introduction

Throughout 40 years of continuous development, assisted reproductive techniques (ARTs) have helped couples overcome infertility through in vitro fertilization (IVF), resulting in more than 9 million ART births worldwide by 2019.^1^ The practice is not without complications, in part owing to the high occurrence of multiple gestations and their inherent complications.^2^ However, previous studies indicate that singletons conceived with ARTs are also more likely to be born preterm,^3^ with low birth weight,^4,5^ and with birth defects^6^ compared with spontaneously conceived children. These perinatal complications have been linked to neurocognitive development impairment^7,8^ and mental health problems later in life.^9,10^ Greater occurrence of rare imprinting disorders such as Angelman and Beckwith-Wiedemann syndromes after ART has also raised concerns that the techniques could disrupt the epigenetic control of chromatin during early embryonic development.^11^

Depression and anxiety are common psychiatric disorders that can lead to considerable problems, and at worst to suicide, which in 2015 was the second leading cause of death among young people globally.^12^ Both conditions can debut in childhood and early adolescence.^13^ Major depression and anxiety tend to cluster within families, seemingly driven by strong genetic components but also by shared family environment.^14,15,16^ Because some researchers argue that emotional stress can contribute to infertility,^17^ depression and anxiety could be more common among children born to couples with infertility,^18^ with ART use further facilitating the transmission of such traits to the next generation. However, the psychiatric health of children and adolescents conceived by ARTs has not been studied extensively,^19^ and previous studies relied on self-reports^20,21^ as well as inappropriate model adjustments, including mediators such as preterm birth.^22^

Because observed elevated risks among children conceived with ARTs could be due to factors associated with the parental underlying infertility rather than the intervention per se, it is vital to disentangle the influence of ARTs from parental characteristics, including infertility and psychiatric health. Furthermore, there is an urgent need to understand the long-term consequences of specific ART procedures, because technical advances such as intracytoplasmic sperm injection (ICSI), prolonged culture of embryos in the laboratory, and freezing and thawing of embryos are now commonly and widely implemented in clinical practice.^23^ With growing numbers of children conceived with ART reaching adolescence and young adulthood, this study was undertaken to advance our understanding of the mental health of this group. In this nationwide, register-based cohort study, we aimed to (1) quantify the occurrence of psychiatric disorders, use of antidepressants, and suicidal behavior among children and adolescents conceived by ARTs and (2) assess whether the association of ARTs with these outcomes spanning across preteen years, adolescence, and early adulthood is driven by parental characteristics associated with the indication for ART use rather than the use per se.

## Methods

### Study Population

**Figure 1.**
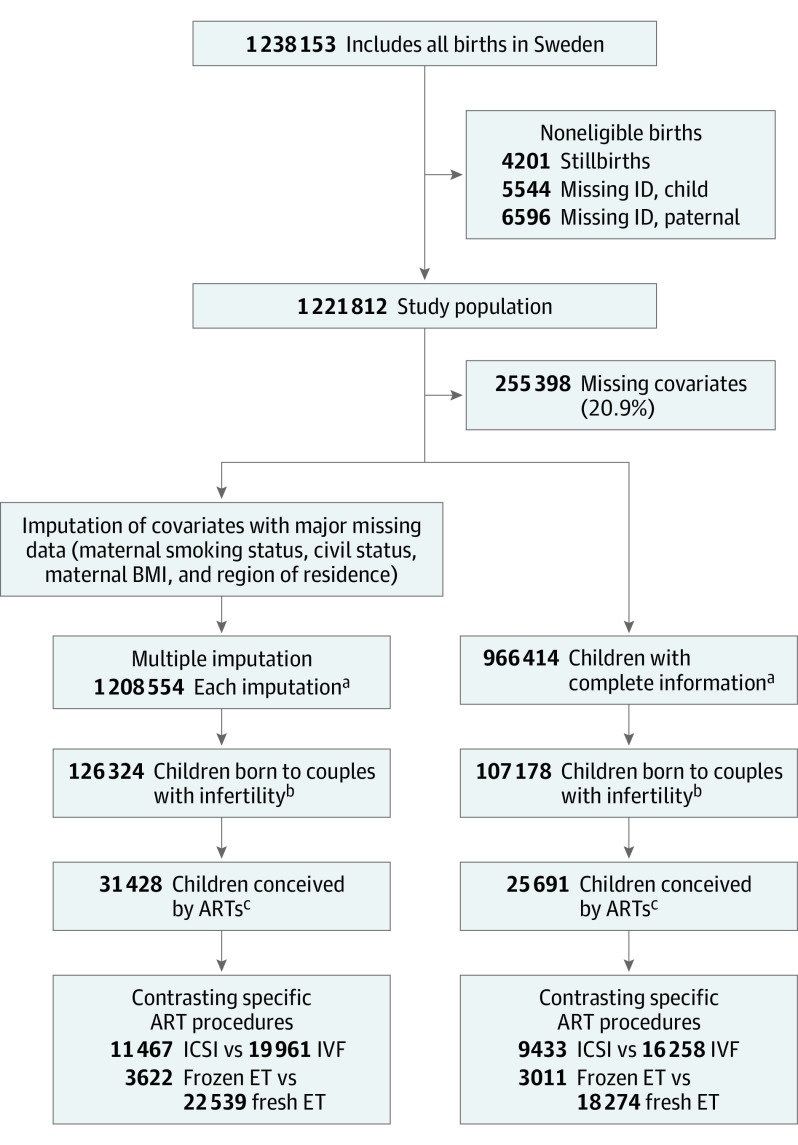
Flowchart of the Study Population ART indicates assisted reproductive technique; BMI, body mass index (calculated as weight in kilograms divided by height in meters squared); ET, embryo transfer; ICSI, intracytoplasmic sperm injection; ID, identification number; and IVF, in vitro fertilization. ^a^Indicates analysis phase 1: participants conceived using ARTs vs the general population. ^b^Indicates analysis phase 2: participants conceived using ARTs vs non-ARTs with infertility. ^c^Indicates analysis phase 3: contrasting specific ART procedures.

This cohort study is based on a linkage of Swedish national registers, enabled by the personal identification number assigned to each individual that allows researchers to track individuals across multiple registers.^24^ The Swedish Medical Birth Register (MBR) was established in 1973 and holds information from standardized forms used to record antenatal and delivery care.^25^ Using this register, we identified a cohort of all children born in Sweden between January 1, 1994, and December 31, 2006 (Figure 1) and followed them up from birth until December 31, 2018, allowing follow-up to a minimum age of 12 years and a maximum age of 25 years. Information on emigration or death was retrieved from the Cause of Death Register and the Register of the Total Population, and fathers were identified using the Swedish Multi-generation Register.^26^ An overview of the information retrieved from the different register sources is given in eTable 1 in the Supplement. The Regional Ethics Committee in Stockholm approved the study and waived the need for individual informed consent, as is customary for register-based studies in Sweden. This study followed the Strengthening the Reporting of Observational Studies in Epidemiology (STROBE) reporting guideline.

### Ascertainment of Parental Fertility Status and ART Use

Since 1982, women included in the MBR have been asked about infertility (defined as taking >1 year of active attempts to achieve pregnancy) in the standard interview at the first antenatal visit. At the same time, the MBR also started collecting information from all IVF clinics in the country to record details on the births conceived with ARTs (use of standard IVF or ICSI or fresh or frozen embryos). From 1995, the enrollment interview also included a follow-up question regarding potential use and type of fertility assistance. The Swedish National Patient Register records diagnoses and procedures from all hospitalizations since 1987 and from specialist outpatient care since 2001.^27^ In both the MBR and National Patient Register, medical conditions are recorded using codes from the *International Classification of Diseases* versions in use.^28^ To optimize exposure ascertainment, we identified parental infertility and potential use of ARTs by combining the information from IVF clinics, diagnostic or procedure coding in the National Patient Register, and maternal self-report in the MBR (eTable 2 in the Supplement). Use of ARTs was grouped into standard IVF and ICSI. Based on the IVF clinic reports, procedures were further grouped according to use of fresh or frozen embryos.

### Follow-up of Offspring Psychiatric Health

Children in the cohort were followed up for the occurrence of psychiatric disorders, suicide behavior, and antidepressant use (eTable 2 in the Supplement). Psychiatric disorders were identified by *International Statistical Classification of Diseases and Related Health Problems, Tenth Revision*, diagnostic codes. Herein, we focused on mood (affective) (F30-F39) and anxiety (F40-F42 and F93) disorders. We further studied major depression (F32 and F33) specifically and separated the anxiety group into obsessive-compulsive disorders (OCD; F42) and all other anxiety disorders (non-OCD; F40, F41, and F93). We considered both suicide attempts and completed suicide with determined intent (X60-X84) from patient records and death certificates. Use of antidepressants (Anatomical Therapeutic Chemical Classification code N06A) was identified using the Swedish Prescribed Drug Register,^29^ which was established in June 2005 and records all dispensed medications outside the hospital, using the Anatomical Therapeutic Chemical Classification System codes. The medications were further categorized as selective serotonin reuptake inhibitor (SSRI) or non-SSRI antidepressants (eTable 2 in the Supplement).^30^ Finally, we also defined severe depression as the combination of a depression diagnosis and dispensation of an antidepressant.

### Parental Background Characteristics

In addition to parental infertility, we considered a wide variety of potential confounding factors that could be determinants of infertility (common causes 0 [CC_0_] in the eFigure in the Supplement) and/or ART use (common causes 1 [CC_1_] in the eFigure in the Supplement), while also influencing the psychiatric health of the offspring. The characteristics included in the study were: (1) parental demographics (maternal and paternal age at delivery, highest level of attained education, and country of origin) obtained from Statistics Sweden; (2) maternal characteristics at the start of pregnancy (parity, region of residence, cohabitation status, smoking, and overweight or obesity); (3) maternal health history (polycystic ovary syndrome and endometriosis); and (4) a summary indicator of parental psychiatric history if either parent had a diagnostic record of a mood disorder or a nonaffective psychosis (mainly relying on hospitalizations, thus capturing more severe conditions). Because this study focused on the overall consequences of ART use, we did not include possible mediating factors such as multiple gestation,^2^ preterm birth,^4^ or low birth weight.^4^

### Statistical Analysis

Data were analyzed from March 17, 2020, to September 10, 2021. First, parental background characteristics were summarized according to parental infertility status and use of ARTs, for which the 3 main and mutually exclusive groups were (1) all children conceived with ARTs; (2) children of couples with infertility who did not use ARTs; and (3) children of couples with no history of infertility (ie, all other children). For the ARTs group, we further considered standard IVF and ICSI separately, and for the subsample with information available, the use of frozen vs fresh embryos. In a similar fashion, we also explored the overall association of parental characteristics with the outcomes of interest.

For each outcome of interest, children were followed up from birth until the first documented occurrence of that outcome, death, emigration, or the end of the study period. For each of the 3 main groups defined by parental infertility and ART use, we first plotted the cumulative incidence of each outcome up to 25 years of age using Kaplan-Meier estimates.

Of all children, 20.9% had missing information in at least 1 covariate of interest. Under the missing random assumption, we conducted multiple imputation with fully conditional specification method.^31^ Given the large sample size, we opted to exclude the few children with missing information on parental educational attainment (<1%) or country of origin (<1%) before imputing all variables with more than 1% missing. The imputed covariates and their corresponding frequency of missing were mother’s region of residence (2.8%), smoking (5.7%), civil status (5.8%), and body mass index (14.8%). In each of the 10 imputation sets, we performed stratified Cox proportional hazards regression with age as underlying time scale and allowing the baseline hazard to vary with birth year. The final estimates were obtained by pooling the results from the imputed data sets using Rubin’s rules.^32^ To explore the association between ARTs and psychiatric outcomes, we first compared children conceived with ARTs with all other children (irrespective of parental infertility status) and then with children whose parents had infertility. After separately comparing either type of fertilization (standard IVF or ICSI) and embryo transfer (fresh or frozen) with the nontreated children born to couples with infertility, we also compared these specific procedures among the treated children only. For all comparisons, we estimated unadjusted associations (crude) and associations with full adjustment, including parental demographics, maternal health conditions, and parental psychiatric history. Schoenfeld residual plots were used to evaluate the proportional hazards assumption for all models. The only noted violation was owing to very few cases of suicidal behavior in young children (younger than 7 years), and, owing to the uncertainty in these reports, we chose to primarily consider suicidal behavior from 12 years or older. In a supplemental analysis, we refitted all models in the sample of children with complete information on covariates (complete-case analysis). We also evaluated specific types of antidepressants used (SSRIs and non-SSRIs) in both multiple imputation and complete-case samples. All analyses were conducted using SAS, version 9.4.4 (SAS Institute Inc) and Stata, version 15.1 (StataCorp LLC).

## Results

Of the 1 221 812 children followed up to a median age of 18 (IQR, 15-21) years, 127 123 (10.4%) were born to couples with infertility and 31 565 (2.6%) were conceived with ARTs (Table 1). Compared with couples with no infertility, mothers in couples experiencing infertility were on average older (eg, ≥40 years, 6345 [5.0%] vs 25 917 [2.4%]) and were more likely to be primiparous (70 531 [55.5%] vs 445 257 [40.7%]), have overweight or obesity (38 405 [30.2%] vs 289 366 [26.4%]), and have been diagnosed with polycystic ovary syndrome (1993 [1.6%] vs 1218 [0.1%]) or endometriosis (2341 [1.8%] vs 2942 [0.3%]). Among couples with infertility, those who received ARTs (IVF or ICSI) were more educated (eg, mothers with postsecondary education, 16 965 [53.7%] vs 44 502 [46.6%]), and the mothers were less likely to smoke (1914 [6.1%] vs 10 827 [11.3%]). Women using ARTs were also more likely to have been diagnosed with polycystic ovary syndrome (715 [2.3%] vs 1278 [1.3%]) or endometriosis (1275 [3.7%] vs 1066 [1.1%]), especially in the group that underwent standard IVF (528 [2.6%] and 976 [4.9%], respectively). Similar to ICSI, the use of frozen embryos became more common over time, but no substantial differences in parental characteristics were noted between those conceived through frozen vs fresh embryos. A complementary exposé of the overall association between parental characteristics and the outcomes under study is provided in eTable 3 in the Supplement.

**Table 1.** Parental Characteristics Among the Study Cohort

Characteristic	Participant group, No. (%)^a^					
	Children of couples with no known infertility (n = 1 094 689)	Children of couples with known infertility				
		Conceived without ARTs (n = 95 558)	Conceived with ARTs			
			Type of ART		Type of embryo transfer^b^	
			IVF (n = 20 067)	ICSI (n = 11 498)	Fresh (n = 22 603)	Frozen (n = 3636)
Year of child’s birth						
1994-1996	278 775 (25.5)	19 353 (20.3)	3702 (18.4)	838 (7.3)	3617 (16.0)	414 (11.4)
1997-1999	229 477 (21.0)	19 687 (20.6)	3936 (19.6)	2854 (24.8)	4952 (21.9)	557 (15.3)
2000-2002	237 583 (21.7)	23 655 (24.8)	4569 (22.8)	2854 (24.8)	5401 (23.9)	581 (16.0)
2003-2006	348 854 (31.9)	32 863 (34.4)	7860 (39.2)	4952 (43.1)	8633 (38.2)	2084 (57.3)
**Paternal characteristics**						
Age at birth, y						
<25	70 306 (6.4)	2789 (2.9)	70 (0.3)	43 (0.4)	49 (0.2)	3 (0.1)
25-29	262 185 (24.0)	17 776 (18.6)	1385 (6.9)	906 (7.9)	1636 (7.2)	180 (5.0)
30-34	386 434 (35.3)	34 292 (35.9)	6432 (32.1)	3557 (30.9)	7217 (31.9)	1087 (29.9)
35-39	239 978 (21.9)	24 749 (25.9)	7132 (35.5)	4013 (34.9)	8108 (35.9)	1311 (36.1)
≥40	135 786 (12.4)	15 952 (16.7)	5048 (25.2)	2979 (25.9)	5593 (24.7)	1055 (29.0)
Highest attained educational level						
Secondary						
Lower	134 244 (12.3)	11 041 (11.6)	1867 (9.3)	1047 (9.1)	2075 (9.2)	321 (8.8)
Upper	563 742 (51.5)	49 976 (52.3)	9113 (45.4)	5483 (47.7)	10 458 (46.3)	1652 (45.4)
Postsecondary	390 908 (35.7)	34 168 (35.8)	9022 (45.0)	4951 (43.1)	10 033 (44.4)	1655 (45.5)
Missing	5795 (0.5)	373 (0.4)	65 (0.3)	17 (0.1)	37 (0.2)	8 (0.2)
Country of birth						
Nordic	913 443 (83.4)	83 488 (87.4)	18 073 (90.1)	10 201 (88.7)	20 355 (90.1)	3303 (90.8)
Other European	68 008 (6.2)	5050 (5.3)	948 (4.7)	636 (5.5)	1130 (5.0)	172 (4.7)
Non-European	112 997 (10.3)	7002 (7.3)	1044 (5.2)	659 (5.7)	1116 (4.9)	161 (4.4)
Unknown	241 (0.02)	18 (0.02)	2 (0.01)	2 (0.02)	2 (0.01)	0
**Maternal characteristics**						
Age at birth, y						
<25	187 449 (17.1)	9389 (9.8)	265 (1.3)	282 (2.5)	302 (1.3)	33 (0.9)
25-29	375 821 (34.3)	29 160 (30.5)	2941 (14.7)	2244 (19.5)	3692 (16.3)	471 (13.0)
30-34	359 377 (32.8)	34 564 (36.2)	8345 (41.6)	4876 (42.4)	9711 (43.0)	1472 (40.5)
35-39	146 125 (13.3)	18 125 (19.0)	7054 (35.2)	3533 (30.7)	7694 (34.0)	1392 (38.3)
≥40	25 917 (2.4)	4320 (4.5)	1462 (7.3)	563 (4.9)	1204 (5.3)	268 (7.4)
Highest educational level attained						
Secondary						
Lower	92 321 (8.4)	6576 (6.9)	955 (4.8)	498 (4.3)	1059 (4.7)	131 (3.6)
Upper	488 940 (44.7)	44 212 (46.3)	8209 (40.9)	4873 (42.4)	9429 (41.7)	1453 (40.0)
Postsecondary	508 071 (46.4)	44 502 (46.6)	10 852 (54.1)	6113 (53.2)	12 087 (53.5)	2047 (56.3)
Missing	5357 (0.5)	268 (0.3)	51 (0.3)	14 (0.1)	28 (0.1)	5 (0.1)
Country of birth						
Nordic	922 685 (84.3)	83 551 (87.4)	17 879 (89.1)	10 113 (88.0)	20 166 (89.2)	3259 (89.6)
Other European	49 048 (4.5)	3972 (4.2)	805 (4.0)	534 (4.6)	966 (4.3)	136 (3.7)
Non-European	119 854 (10.9)	7905 (8.3)	1367 (6.8)	849 (7.4)	1463 (6.5)	239 (6.6)
Unknown	3102 (0.3)	130 (0.1)	16 (0.1)	2 (0.02)	8 (0.04)	2 (0.1)
Region of residence in Sweden						
East	346 953 (31.7)	30 460 (31.9)	7725 (38.5)	3450 (30.0)	7834 (34.7)	1137 (31.3)
Central	246 278 (22.5)	19 925 (20.9)	4017 (20.0)	2204 (19.2)	4580 (20.3)	706 (19.4)
North	85 346 (7.8)	6868 (7.2)	1194 (6.0)	871 (7.6)	1435 (6.3)	192 (5.3)
South	163 551 (14.9)	17 866 (18.7)	3151 (15.7)	2144 (18.6)	4044 (17.9)	453 (12.5)
West	214 837 (19.6)	18 907 (19.8)	3849 (19.2)	2749 (23.9)	4604 (20.4)	1136 (31.2)
Missing	37 724 (3.4)	1532 (1.6)	131 (0.7)	80 (0.7)	106 (0.5)	12 (0.3)
Parity						
1	445 257 (40.7)	51 122 (53.5)	12 220 (60.9)	7189 (62.5)	14 500 (64.2)	1900 (52.3)
2	407 524 (37.2)	31 160 (32.6)	6081 (30.3)	3428 (29.8)	6429 (28.4)	1327 (36.5)
3	165 230 (15.1)	9851 (10.3)	1384 (6.9)	720 (6.3)	1325 (5.9)	327 (9.0)
≥4	76 678 (7.0)	3425 (3.6)	382 (1.9)	161 (1.4)	349 (1.5)	82 (2.3)
Smoking						
Yes	128 043 (11.7)	10 827 (11.3)	1325 (6.6)	589 (5.1)	1352 (6.0)	181 (5.0)
Missing	65 988 (6.0)	2452 (2.6)	1382 (6.9)	823 (7.2)	1689 (7.5)	254 (7.0)
Overweight or obese						
Yes	289 366 (26.4)	29 194 (30.6)	5580 (27.8)	3631 (31.6)	6593 (29.2)	1023 (28.1)
Missing	164 958 (15.1)	9977 (10.4)	2989 (14.9)	1594 (13.9)	3428 (15.2)	510 (14.0)
Civil status						
Cohabitating	974 335 (89.0)	91 099 (95.3)	18 595 (92.7)	10 691 (93.0)	20 904 (92.5)	3369 (92.7)
Living alone	53 053 (4.8)	2383 (2.5)	144 (0.7)	82 (0.7)	104 (0.5)	30 (0.8)
Missing	67 301 (6.1)	2076 (2.2)	1328 (6.6)	725 (6.3)	1595 (7.1)	237 (6.5)
Maternal medical conditions						
PCOS	1218 (0.1)	1278 (1.3)	528 (2.6)	187 (1.6)	495 (2.2)	89 (2.4)
Endometriosis	2942 (0.3)	1066 (1.1)	976 (4.9)	299 (2.6)	963 (4.3)	158 (4.3)
Parental psychiatric history^c^						
Mood disorders	26 949 (2.5)	2458 (2.6)	549 (2.7)	265 (2.3)	580 (2.6)	82 (2.3)
Psychosis	3561 (0.3)	294 (0.3)	42 (0.2)	32 (0.3)	44 (0.2)	8 (0.2)

Abbreviations: ARTs, assisted reproductive techniques; ICSI, intracytoplasmic sperm injection; IVF, in vitro fertilization; PCOS, polycystic ovary syndrome.

^a^ Percentages have been rounded and may not total 100.

^b^ Concerns a subset (n = 26 239) of participants for whom type of embryo transfer was recorded.

^c^ Summary indicator of either parent having a diagnosis.

**Figure 2.**
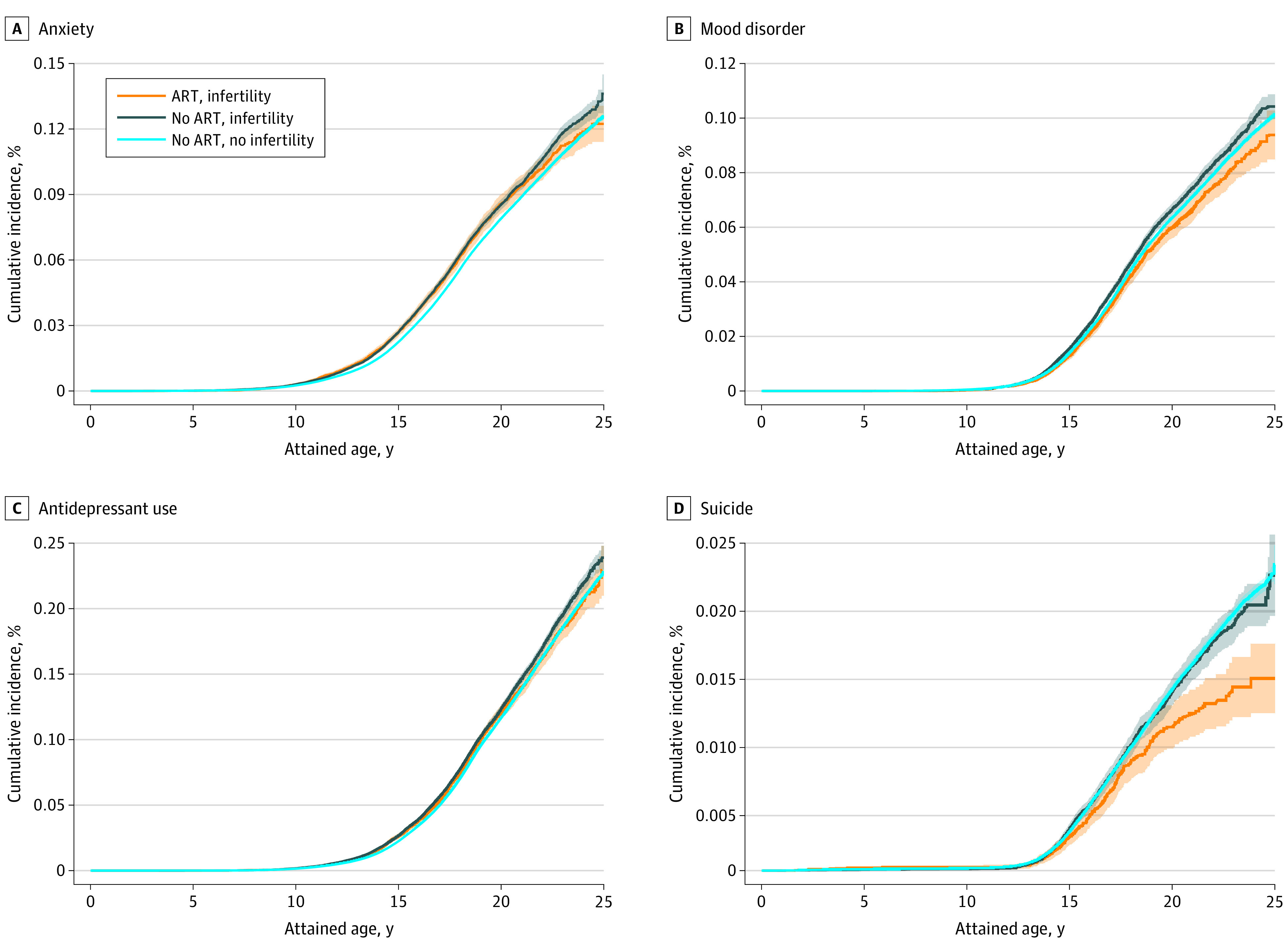
Estimated Cumulative Incidence of Indices of Psychiatric Health to 25 Years of Age, According to Parental Infertility and Use of Assisted Reproductive Techniques (ARTs) Shaded areas indicate 95% CIs.

Among children born to couples without infertility, the estimated cumulative incidence at the end of puberty (18 years of age) was 5.6% for anxiety, 0.9% for complete or attempted suicide, and 7.0% for antidepressant use (Figure 2). The cumulative incidence of mood disorders was 4.4%, with depression accounting for the vast majority (4.2%; specific plot available at request). Among children conceived with ARTs, the corresponding cumulative incidences were overall comparable or lower across all ages. Children of couples with infertility who were not conceived with ARTs were noted to be at slightly elevated overall risk of anxiety (6.2% [95% CI, 6.0%-6.4%]) and mood disorders (4.7% [95% CI, 4.5%-4.9%]) (Figure 2).

In a regression comparison with all other children (irrespective of parental infertility), children conceived with ARTs were not at any disadvantage with respect to the studied outcomes (Table 2), except for a slightly elevated rate of anxiety (hazard ratio [HR], 1.07 [95% CI, 1.02-1.12]), primarily driven by OCD (HR, 1.35 [95% CI, 1.20-1.51]). Although adjustment for parental characteristics attenuated observed inverse associations with mood disorder and suicide behavior, as well as a substantial part of the association with OCD (adjusted HR [aHR], 1.10 [95% CI, 0.98-1.24]), it had no influence on the small difference in anxiety overall (aHR, 1.08 [95% CI, 1.03-1.13]). This was furthermore matched by a slightly elevated rate of antidepressant use (aHR, 1.05 [95% CI, 1.01-1.09]). When the comparison was restricted to children of couples with known infertility, however, no differences were seen for OCD (aHR, 1.02 [95% CI, 0.89-1.17]) or any of the other outcomes in adjusted models (Table 2). In further evaluation of specific ART procedures, children conceived with ICSI were no different from children of couples with infertility conceived without ARTs, or by standard IVF (Table 3). Children conceived with fresh, but not frozen, embryo transfer had a lower risk of mood disorders compared with children of couples with infertility conceived without ARTs (aHR, 0.90 [95% CI, 0.83-0.97]). In the direct contrast of the 2 procedures, frozen embryo transfer appeared associated with elevated risk of mood disorders (aHR, 1.25 [95% CI, 1.03-1.52]) and antidepressant use (aHR, 1.16 [95% CI, 1.01-1.33]). Finally, the complete cases analysis revealed similar but somewhat attenuated estimates with wider CIs (eTables 4 and 5 in the Supplement), and the evaluation of SSRI and non-SSRI use showed similar results (eTables 6 and 7 in the Supplement).

**Table 2.** Association Between Assisted Reproductive Techniques and Indices of Psychiatric Health

Indices of psychiatric health	No. of events by group		HR (95% CI)	
	Exposed	Nonexposed	Crude^a^	Adjusted^b^
**ARTs-conceived children compared with all other children**				
Anxiety	1725	71 887	1.07 (1.02-1.12)	1.08 (1.03-1.13)
OCD	307	9782	1.35 (1.20-1.51)	1.10 (0.98-1.24)
Non-OCD	1557	67 104	1.05 (1.00-1.10)	1.07 (1.02-1.13)
Mood disorder	1142	55 555	0.93 (0.88-0.99)	0.99 (0.93-1.05)
Depression	1075	51 997	0.94 (0.88-1.00)	0.98 (0.92-1.04)
Severe depression^c^	933	44 727	0.93 (0.87-0.99)	0.98 (0.92-1.05)
Suicide	196	11 733	0.77 (0.66-0.89)	1.03 (0.89-1.19)
Antidepressant use	2383	109 360	1.03 (0.99-1.07)	1.05 (1.01-1.09)
**ARTs-conceived children compared with non–ARTs-conceived children with parental infertility**				
Anxiety	1725	5931	0.98 (0.93-1.03)	1.02 (0.96-1.08)
OCD	307	890	1.14 (1.00-1.30)	1.02 (0.89-1.17)
Non-OCD	1557	5499	0.96 (0.91-1.02)	1.01 (0.95-1.07)
Mood disorder	1142	4386	0.89 (0.83-0.95)	0.95 (0.89-1.02)
Depression	1075	4114	0.89 (0.83-0.95)	0.95 (0.89-1.02)
Severe depression^c^	933	3566	0.88 (0.82-0.95)	0.94 (0.87-1.01)
Suicide	196	879	0.77 (0.66-0.90)	0.92 (0.78-1.08)
Antidepressant use	2383	8620	0.95 (0.91-0.99)	1.00 (0.95-1.05)

Abbreviations: ARTs, assisted reproductive techniques; HR, hazard ratio; OCD, obsessive-compulsive disorder.

^a^ The Cox proportional hazards regression model was stratified by birth year.

^b^ The model was adjusted for parents’ age at delivery, country of origin, highest educational attainment, parity, civil status, region of residence, maternal smoking status, maternal overweight and obesity, maternal medical conditions, and parental psychiatric history.

^c^ Diagnosis of depression combined with antidepressant use.

**Table 3.** Association Between Specific Assisted Reproductive Techniques and Indices of Psychiatric Health

Psychiatric diagnosis	Adjusted HR (95% CI)^a^			
	Type of fertilization		Type of embryo transfer	
	IVF	ICSI	Fresh	Frozen
**ARTs compared with non-ARTs with parental infertility**				
Anxiety	1.00 (0.94-1.07)	1.05 (0.96-1.15)	0.98 (0.92-1.04)	1.08 (0.92-1.26)
OCD	0.97 (0.83-1.14)	1.11 (0.91-1.35)	1.02 (0.88-1.19)	1.14 (0.80-1.61)
Non-OCD	1.00 (0.93-1.07)	1.04 (0.95-1.14)	0.97 (0.91-1.04)	1.02 (0.86-1.21)
Mood disorder	0.95 (0.88-1.03)	0.94 (0.84-1.05)	0.90 (0.83-0.97)	1.07 (0.89-1.29)
Depression	0.95 (0.87-1.03)	0.94 (0.84-1.05)	0.90 (0.83-0.98)	1.09 (0.90-1.32)
Severe depression	0.95 (0.87-1.04)	0.91 (0.81-1.03)	0.90 (0.83-0.98)	1.04 (0.84-1.28)
Suicide	0.95 (0.79-1.14)	0.87 (0.67-1.14)	0.91 (0.76-1.09)	0.92 (0.56-1.51)
Antidepressant drug use	0.97 (0.92-1.03)	1.05 (0.97-1.13)	0.97 (0.92-1.02)	1.09 (0.96-1.24)
**ART procedures directly compared**				
Anxiety	1 [Reference]	1.02 (0.92-1.13)	1 [Reference]	1.12 (0.95-1.32)
OCD	1 [Reference]	1.08 (0.86-1.36)	1 [Reference]	1.13 (0.79-1.62)
Non-OCD	1 [Reference]	1.02 (0.92-1.14)	1 [Reference]	1.08 (0.91-1.29)
Mood disorder	1 [Reference]	1.01 (0.89-1.15)	1 [Reference]	1.25 (1.03-1.52)
Depression	1 [Reference]	1.01 (0.89-1.15)	1 [Reference]	1.28 (1.05-1.57)
Severe depression	1 [Reference]	0.97 (0.84-1.12)	1 [Reference]	1.24 (1.00-1.54)
Suicide	1 [Reference]	0.91 (0.65-1.25)	1 [Reference]	1.06 (0.63-1.80)
Antidepressant drug use	1 [Reference]	1.06 (0.97-1.16)	1 [Reference]	1.16 (1.01-1.33)

Abbreviations: ART, assisted reproductive technique; HR, hazard ratio; ICSI, intracytoplasmic sperm injection; IVF, in vitro fertilization; OCD, obsessive-compulsive disorder.

^a^ The model was adjusted for birth year, parents’ age at delivery, country of origin, highest educational attainment, parity, civil status, region of residence, maternal smoking status, maternal overweight and obesity, maternal medical conditions, and parental psychiatric history.

## Discussion

Overall, we found that adolescents conceived with ARTs had a slightly higher risk of anxiety and antidepressant use but a lower risk of mood disorder and suicidal behavior compared with all other children. Importantly, the observed differences in risk were explained by differences in parental characteristics, including the underlying infertility, rather than the ART intervention itself. Type of fertilization (standard IVF or ICSI) had no association with outcomes. Fresh but not frozen embryo transfer was associated with a lower risk of mood disorders when compared with children from couples with infertility who conceived without ARTs, making frozen embryo transfer appear less advantageous in direct comparison with fresh embryo transfer.

This nationwide register-based cohort study enabled investigation of psychiatric health outcomes based on clinical diagnosis and medication use, with follow-up into adolescence and early adulthood. Children conceived with ART were found to be at a slightly elevated risk of anxiety compared with all other children, largely driven by an excessive risk of OCD. After adjustment for parental characteristics, children conceived with ART were also slightly more likely to be prescribed antidepressants. At present, SSRIs are still the most commonly prescribed class of antidepressants for both depression and anxiety,^33^ whereas prescription of non-SSRI antidepressants might reflect a more severe depressive disorder.^30^ However, the slightly elevated risks were no longer seen when the comparison was made only among individuals whose parents experienced infertility. For mood disorders and suicidal behavior, adolescents conceived with ART were if anything at lower risk compared with all other adolescents, and this appeared to be explained by differences in parental background characteristics. Depression, which accounted for most mood disorders in children and adolescents, consequentially followed the same pattern. Before our study, the research on psychiatric disorders in children conceived with ART was scarce and findings were conflicting. An early study that relied on self-reported outcomes^20^ raised concerns for depression and anxiety levels among children conceived with ART, but the findings could not be reproduced in a follow-up study.^21^ Scanning any mental disorders (*ICD-10* codes F00-F99), a recent study in Finland^22^ followed up 17 610 singletons conceived using ART and found a higher risk of any psychiatric diagnosis compared with the general population. However, the results for depression as well as anxiety were underpowered, and interpretation was complicated by the control for prematurity, which could be a mediator of a potential effect of ART. Last but most important, none of previous studies disentangled the potential influence of the fertility treatment from the underlying infertility, which has been shown to be associated with poorer mental health outcomes.^18^

This long- and wide-spanning birth cohort also enabled exploration of the association of outcomes with different ARTs. When compared with spontaneously conceived children of couples with infertility, we found no substantial differences in the studied outcomes for children conceived by ICSI, standard IVF, or frozen embryo transfer, but observed a lower risk of mood disorder for children conceived by fresh embryo transfer. In the direct comparison of types of embryo transfer, the group conceived with frozen embryo thus appeared at elevated risk of depression and use of antidepressants. Whether the findings could be due to a beneficial causal effect or perhaps more plausibly unmeasured confounding, we note that they raise no concerns for the procedures’ potential influence on psychiatric health. In previous contrasts, frozen embryo transfer has appeared more favorable with respect to preterm birth and low birth weight,^34^ whereas a recent Swedish study^3^ found it associated with higher mortality in the first year of life. We note that as the technology has improved over time, with vitrification replacing slow freezing, so have the indications for use, with elective freeze-all for later transfer being advocated in specific clinical situations. It will thus be necessary to continue to monitor the outcomes of children born from frozen embryo transfer.

Prospectively gathered data in national registers allowed thorough consideration of parental background characteristics and comprehensive identification of ART use from specific IVF-clinic reports, general medical records (diagnostic or procedure codes), and maternal self-report. Because the degree of coverage from either source varies, but each is expected to contain few false-positive findings, we combined all sources to boost sensitivity without compromising specificity. Although infertility was primarily based on mothers’ response to questions about involuntary childlessness and time to pregnancy at enrollment in antenatal care, this information was further complemented with potential clinical diagnosis of infertility.

### Limitations

Our study also has some limitations. Anxiety and mood disorders are not typically childhood-onset disorders, and our study with follow-up mostly focusing on adolescence is uninformative as to whether depression and anxiety might be more common among offspring in adulthood. Second, the degree of missing information was moderate, and it concerned several of the relevant covariates in the cohort. Under the assumption of missing at random, we performed multiple imputations to maximize the power in the main analysis. Our findings reflect ART practices in Sweden around the millennium, and the generalizability to other settings and time periods should consider differences in clinical practice. In Sweden, the occurrence of multiple gestations decreased substantially after the implementation of the single-embryo transfer policy in the early 2000s.^35^ Aside from increased reliance on ICSI without male factor infertility, the use of frozen embryos has increased dramatically in recent decades thanks to improvements related to vitrification compared with slow freezing.

## Conclusions

In this follow-up of a Swedish national birth cohort study, the findings were reassuring with respect to the psychiatric health of adolescents conceived with ARTs. An elevated risk of OCD in the overall comparison with all other adolescents may be explained by differences in parental characteristics, and parental use of ARTs could be a candidate in potential screening of this disorder.

## Notes

Supplement. **eFigure.** Illustration of Potential Pathways Linking Parental Infertility and Use of ART With Psychiatric Outcomes in Children **eTable 1.** Overview of Information Used in the Analysis **eTable 2.** Variable Definitions and Their Sources **eTable 3.** Parental Characteristics’ Association With the Psychiatric Indices **eTable 4.** Association Between ART and Indices of Psychiatric Health in Individuals With Complete Information **eTable 5.** Association Between Specific ART Procedures and Indices of Psychiatric Health in Individuals With Complete Information **eTable 6.** Association Between ART and Different Antidepressant Use in Individuals After Multiple Imputation or Complete Information **eTable 7.** Association Between ART Specific Procedures and Different Antidepressant Use in Individuals After Multiple Imputation or Complete Information
