# Supplementary material for: Long-term Follow-up of Psychiatric Disorders in Children and Adolescents Conceived by Assisted Reproductive Techniques in Sweden
Source: JAMA Psychiatry. 2021 Dec 15;79(2):1–10. doi: 10.1001/jamapsychiatry.2021.3647 (PMC8674804; doi:10.1001/jamapsychiatry.2021.3647)
Supplement: Supplement. — eFigure. Illustration of Potential Pathways Linking Parental Infertility and Use of ART With Psychiatric Outcomes in Children eTable 1. Overview of Information Used in the Analysis eTable 2. Variable Definitions and Their Sources eTable 3. Parental Characteristics’ Association With the Psychiatric Indices eTable 4. Association Between ART and Indices of Psychiatric Health in Individuals With Complete Information eTable 5. Association Between Specific ART Procedures and Indices of Psychiatric Health in Individuals With Complete Information eTable 6. Association Between ART and Different Antidepressant Use in Individuals After Multiple Imputation or Complete Information eTable 7. Association Between ART Specific Procedures and Different Antidepressant Use in Individuals After Multiple Imputation or Complete Information [file jamapsychiatry-e213647-s001.pdf]

Supplementary Online Content

Wang C, Johansson ALV, Rodriguez-Wallberg KA, et al. Long-term follow-up of psychiatric disorders in children and adolescents conceived by assisted reproductive techniques in Sweden. *JAMA Psychiatry*. Published online December 15, 2021. doi:10.1001/jamapsychiatry.2021.3647

eFigure. Illustration of Potential Pathways Linking Parental Infertility and Use of ART With Psychiatric Outcomes in Children
eTable 1. Overview of Information Used in the Analysis
eTable 2. Variable Definitions and Their Sources
eTable 3. Parental Characteristics' Association With the Psychiatric Indices
eTable 4. Association Between ART and Indices of Psychiatric Health in Individuals With Complete Information
eTable 5. Association Between Specific ART Procedures and Indices of Psychiatric Health in Individuals With Complete Information
eTable 6. Association Between ART and Different Antidepressant Use in Individuals After Multiple Imputation or Complete Information
eTable 7. Association Between ART Specific Procedures and Different Antidepressant Use in Individuals After Multiple Imputation or Complete Information

This supplementary material has been provided by the authors to give readers additional information about their work.

© 2021 Wang C et al. *JAMA Psychiatry.*

eFigure. Illustration of Potential Pathways Linking Parental Infertility and Use of ART With Psychiatric Outcomes in Children

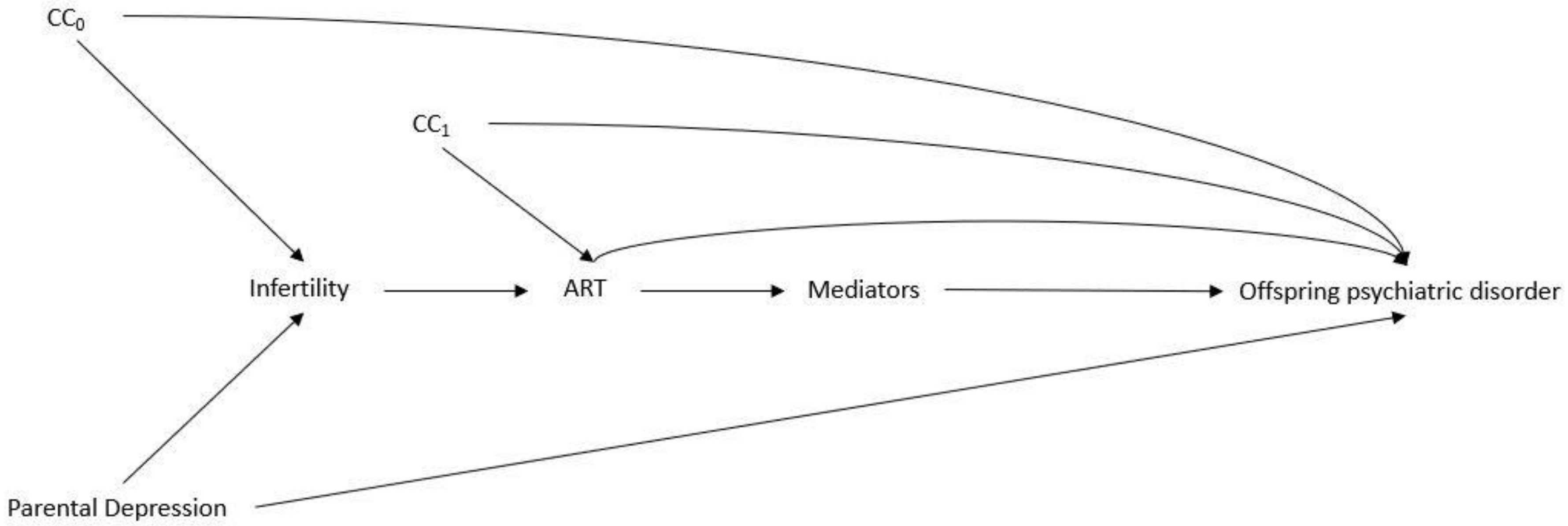

© 2021 Wang C et al. *JAMA Psychiatry.*

eTable 1. Overview of Information Used in the Analysis

Variable	Source	Definition
**Exposure**		
ART use	MBR[a] (maternal and IVF-clinic report), NPR[b]	IVF or IVF combined with ICSI, fresh or frozen-thawed embryo transfer
**Outcome**		
Mood disorder	NPR	Major depressive disorder and other affective disorders
Anxiety	NPR	OCD[c] and non-OCD
Antidepressant drug use	PDR[d]	SSRI[e] and non-SSRI
Suicide	NPR, CDR[f]	Suicidal attempts and completed suicide
**Covariates**		
Parental infertility	MBR (maternal and IVF-clinic report), NPR	Infertility by maternal report, clinical diagnosis in either parent, or record of ART use
Year at birth	MBR	Continuous
Parental education	SCB[g]	Lower secondary, Upper secondary, Post secondary
Parental country of birth	SCB	Nordic, other European, non-European
Paternal age at birth	SCB	Less than 25, 25-29,30-34, 35-39, 40 or over 40
Maternal age at birth	MBR	Less than 25, 25-29,30-34, 35-39, 40 or over 40
Mother region of residence	SCB	East, mid, south, west, north
Parity	MBR	1(=no previous child), 2, 3, 4 and more
Maternal smoking early pregnancy	MBR	Yes/No
Maternal BMI early pregnancy	MBR	<26, ≥26 (overweight)
Maternal medical conditions	NPR	PCOS[h] or Endometriosis
Parental psychiatric history	NPR	Mood disorders or Non-affective psychosis

a: Medical Birth Register; b: National Patient Register; c: Obsessive-Compulsive Disorders; d: Prescribed Drug Register; e: Selective serotonin reuptake inhibitors; f: Cause of Death Register; g: Statistic Sweden; h: Polycystic Ovary Syndrome.

© 2021 Wang C et al. *JAMA Psychiatry.*

eTable 2. Variable Definitions and Their Sources

		MBR[a]	NPR[b], MBR[a]				PDR[c]	Other Register
		Self-report	ICD8	ICD9	ICD 10	Procedure	ATC	Variables
Exposure	Infertility in female		628	628	N97			IVF-clinic 1982-2006; Quality IVF register 2007-
	Infertility in male		606	606	N46			
	Infertility NOS	since 1982, time-to-pregnancy >=1 year		V23A	Z35.0			
	IVF	since 1995, yes/no			Z31.2<br>O26.8A<br>Z31.3<br>Z31.2A<br>Z31.2C<br>Z31.2B	LCA30<br>DL002<br>DL003<br>DL001<br>DL007		
	ICSI	since 1999, yes/no			Z31.2B	DL001<br>DL007		
Outcome	Mood disorder				F30-F39			
	Major depression				F32, F33			
	Anxiety				F40, F41, F42 (OCD), F93			
	Antidepressant drugs						N06A (SSRI: N06AB)	
Outcome	Suicide (attempt and complete)				X60-X84			X60-X84 in Cause of Death Register

© 2021 Wang C et al. *JAMA Psychiatry.*

		MBR[a]	NPR[b], MBR[a]				PDR[c]	Other Register
		**Self-report**	**ICD8**	**ICD9**	**ICD 10**	**Procedure**	**ATC**	**Variables**
Other covariates								
Parental psychiatric history (Mood disorder and Non-affective psychosis)			295-299<br>300.4	295-299<br>311<br>300E	F20-F39			
Pre-existing conditions								
	PCOS		256.9	256E	E28.2			
	Endometriosis		625.3	617	N80			

a: Medical Birth Register; b: National Patient Register, c: Prescribed Drug Register

© 2021 Wang C et al. *JAMA Psychiatry.*

eTable 3. Parental Characteristics' Association With the Psychiatric Indices

	Crude hazard ratios (95% Confidence intervals)			
	**Mood disorder**	**Anxiety**	**Antidepressants**	**Suicide**
**Year at birth**				
1994-1996	Ref	Ref	Ref	Ref
1997-1999	1.24 (1.21, 1.27)	1.39 (1.36, 1.42)	1.28 (1.26, 1.30)	1.07 (1.02, 1.12)
2000-2002	1.62 (1.58, 1.67)	2.03 (1.98, 2.08)	1.69 (1.65, 1.72)	1.14 (1.07, 1.21)
2003-2006	2.22 (2.13, 2.31)	2.93 (2.84, 3.03)	2.39 (2.32, 2.47)	1.28 (1.17, 1.41)
**Paternal characteristics**				
Age at birth				
less than 25	1.38 (1.33, 1.43)	1.37 (1.32, 1.41)	1.30 (1.27, 1.34)	1.63 (1.52, 1.75)
25-29	Ref	Ref	Ref	Ref
30-34	0.94 (0.92, 0.96)	0.98 (0.96, 1.00)	0.95 (0.93, 0.96)	0.83 (0.79, 0.88)
35-39	0.97 (0.94, 0.99)	1.03 (1.01, 1.06)	0.97 (0.95, 0.99)	0.88 (0.83, 0.93)
40 or over	1.04 (1.01, 1.07)	1.11 (1.08, 1.15)	1.05 (1.03, 1.07)	0.98 (0.92, 1.05)
Highest education				
Lower secondary	Ref	Ref	Ref	Ref
Upper secondary	0.93 (0.90, 0.96)	0.90 (0.87, 0.92)	0.94 (0.92, 0.96)	0.74 (0.71, 0.79)
Post-secondary	0.86 (0.83, 0.88)	0.81 (0.79, 0.83)	0.87 (0.85, 0.88)	0.53 (0.50, 0.56)
Country of birth				
Nordic	Ref	Ref	Ref	Ref
Other European	0.73 (0.70, 0.77)	0.86 (0.83, 0.90)	0.73 (0.71, 0.76)	0.89 (0.81, 0.99)
Non-European	0.76 (0.73, 0.79)	0.82 (0.79, 0.85)	0.69 (0.67, 0.71)	1.08 (1.00, 1.17)
**Maternal characteristics**				
Age at birth				
less than 25	1.29 (1.26, 1.33)	1.27 (1.24, 1.30)	1.22 (1.20, 1.24)	1.61 (1.53, 1.70)
25-29	Ref	Ref	Ref	Ref
30-34	0.99 (0.97, 1.02)	1.03 (1.01, 1.05)	0.99 (0.98, 1.01)	0.92 (0.87, 0.97)
35-39	1.06 (1.03, 1.10)	1.15 (1.12, 1.18)	1.08 (1.06, 1.10)	1.03 (0.96, 1.10)
40 or over	1.19 (1.12, 1.27)	1.32 (1.25, 1.38)	1.18 (1.12, 1.23)	1.24 (1.09, 1.42)
Highest education				
Lower secondary	Ref	Ref	Ref	Ref
Upper secondary	0.86 (0.83, 0.89)	0.81 (0.79, 0.83)	0.88 (0.86, 0.90)	0.65 (0.61, 0.69)
Post-secondary	0.84 (0.81, 0.87)	0.75 (0.73, 0.77)	0.85 (0.83, 0.87)	0.47 (0.44, 0.50)

© 2021 Wang C et al. *JAMA Psychiatry.*

	**Crude hazard ratios (95% Confidence intervals)**			
	**Mood disorder**	**Anxiety**	**Antidepressants**	**Suicide**
Country of birth				
Nordic	Ref	Ref	Ref	Ref
Other European	0.69 (0.65, 0.74)	0.79 (0.75, 0.83)	0.69 (0.66, 0.72)	0.82 (0.73, 0.93)
Non-European	0.66 (0.63, 0.69)	0.71 (0.68, 0.74)	0.59 (0.57, 0.61)	0.88 (0.81, 0.96)
Region of residence				
East	Ref	Ref	Ref	Ref
Middle	0.91 (0.89, 0.93)	0.86 (0.84, 0.87)	0.92 (0.91, 0.94)	0.94 (0.89, 0.99)
North	0.98 (0.94, 1.01)	0.81 (0.79, 0.84)	1.02 (1.00, 1.05)	0.96 (0.89, 1.04)
South	0.70 (0.68, 0.72)	0.76 (0.74, 0.78)	0.89 (0.87, 0.91)	0.70 (0.66, 0.75)
West	0.83 (0.81, 0.85)	0.76 (0.75, 0.78)	0.95 (0.93, 0.97)	0.78 (0.74, 0.83)
Parity				
1	Ref	Ref	Ref	Ref
2	0.96 (0.94, 0.98)	0.91 (0.90, 0.93)	0.95 (0.94, 0.97)	1.12 (1.07, 1.17)
3	1.00 (0.98, 1.03)	0.95 (0.93, 0.97)	0.99 (0.97, 1.01)	1.26 (1.19, 1.34)
4 and more	1.13 (1.09, 1.18)	1.11 (1.07, 1.14)	1.12 (1.09, 1.15)	1.73 (1.61, 1.86)
Civil status				
Co-habitating	Ref	Ref	Ref	Ref
Living alone	1.62 (1.56, 1.68)	1.61 (1.56, 1.66)	1.49 (1.45, 1.53)	2.28 (2.13, 2.44)
Smoking	1.53 (1.49, 1.56)	1.49 (1.46, 1.52)	1.47 (1.44, 1.49)	2.26 (2.16, 2.37)
Overweight or Obese	1.17 (1.15, 1.19)	1.13 (1.11, 1.15)	1.12 (1.10, 1.14)	1.24 (1.18, 1.29)
**Maternal medical conditions**				
PCOS	1.52 (1.23, 1.87)	1.64 (1.38, 1.94)	1.49 (1.28, 1.74)	1.09 (0.63, 1.87)
Endometriosis	1.61 (1.40, 1.87)	1.54 (1.36, 1.75)	1.59 (1.42, 1.77)	1.48 (1.06, 2.06)
**Parental psychiatric history**				
Mood disorders	2.16 (2.07, 2.26)	2.16 (2.07, 2.25)	2.01 (1.94, 2.08)	2.64 (2.42, 2.89)
Non affective psychosis	1.77 (1.54, 2.04)	1.92 (1.71, 2.16)	1.67 (1.51, 1.85)	2.85 (2.24, 3.61)

© 2021 Wang C et al. *JAMA Psychiatry.*

eTable 4. Association Between ART and Indices of Psychiatric Health in Individuals With Complete Information

		Number of Events (exposed/non-exposed)	Crude hazard ratio[a] (lower CI, upper CI)	Adjusted hazard ratio[b] (lower CI, upper CI)
**ART compared to all other**				
	Anxiety	1370/56514	0.99 (0.94, 1.04)	1.04 (0.98, 1.10)
	OCD	242/7752	**1.28 (1.13, 1.45)**	1.10 (0.96, 1.25)
	Non-OCD	1229/52699	0.95 (0.90, 1.01)	1.02 (0.97, 1.08)
	Mood disorder	908/43667	**0.88 (0.82, 0.94)**	0.96 (0.90, 1.03)
	Depression	836/40901	**0.88 (0.82, 0.94)**	0.96 (0.90, 1.03)
	Severe depression[c]	748/35146	**0.90 (0.84, 0.97)**	0.98 (0.91, 1.06)
	Suicide	144/9095	**0.70 (0.60, 0.83)**	0.94 (0.79, 1.11)
	Antidepressant drug use	1865/85888	0.98 (0.93, 1.02)	1.04 (0.99, 1.09)
**ART compared to those non ART with parental infertility**				
	Anxiety	1370/5047	**0.93 (0.88, 0.99)**	0.99 (0.93, 1.05)
	OCD	242/792	1.11 (0.96, 1.28)	1.00 (0.86, 1.16)
	Non-OCD	1229/4672	**0.91 (0.85, 0.97)**	0.98 (0.92, 1.05)
	Mood disorder	908/3766	**0.85 (0.79, 0.91)**	**0.92 (0.85, 0.99)**
	Depression	836/3709	**0.85 (0.79, 0.92)**	0.92 (0.85, 1.00)
	Severe depression[c]	748/3063	**0.86 (0.80, 0.94)**	0.93 (0.85, 1.01)
	Suicide	144/749	**0.70 (0.58, 0.83)**	0.83 (0.69, 1.01)
	Antidepressant drug use	1865/7696	**0.93 (0.89, 0.98)**	0.99 (0.94, 1.05)

ART is assisted reproductive techniques CI is confidence interval (95%) OCD is obsessive compulsive disorder

a. The model was stratified Cox proportional hazard regression was stratified by birth year.
b. The model was also adjusted for parents' age at delivery, origin, highest education, parity, civil status, region of residence, maternal smoking status, maternal overweight and obesity, maternal medical conditions, and parental psychiatric history.
c. Diagnosis of depression combined with antidepressant use

© 2021 Wang C et al. *JAMA Psychiatry.*

eTable 5. Association Between Specific ART Procedures and Indices of Psychiatric Health in Individuals With Complete Information

		Adjusted hazard ratioa (lower CI, upper CI)			
		**Type of fertilization**		**Type of embryo transfer**	
		**IVF**	**ICSI**	**Fresh**	**Frozen**
**ART specific procedures compared to those non ART with parental infertility**					
	Anxiety	1.01 (0.94, 1.08)	0.94 (0.86, 1.04)	0.96 (0.90, 1.03)	0.98 (0.83, 1.17)
	OCD	0.98 (0.82, 1.17)	1.05 (0.84, 1.32)	1.01 (0.85, 1.19)	1.15 (0.79, 1.67)
	Non-OCD	1.00 (0.93, 1.08)	0.93 (0.83, 1.03)	0.95 (0.88, 1.02)	0.91 (0.75, 1.11)
	Mood disorder	0.95 (0.87, 1.03)	0.85 (0.75, 0.96)	0.89 (0.81, 0.97)	0.99 (0.80, 1.23)
	Depression	0.95 (0.86, 1.04)	0.85 (0.75, 0.97)	0.89 (0.81, 0.97)	1.01 (0.81, 1.25)
	Severe depression	0.95 (0.86, 1.05)	0.87 (0.76, 1.00)	0.89 (0.81, 0.98)	0.99 (0.78, 1.25)
	Suicide	0.87 (0.70, 1.08)	0.77 (0.56, 1.05)	0.82 (0.67, 1.02)	0.71 (0.38, 1.33)
	Antidepressant drug use	0.99 (0.93, 1.05)	0.99 (0.91, 1.08)	0.98 (0.92, 1.04)	1.06 (0.91, 1.22)
**ART specific procedures directly compared**					
	Anxiety	Ref	0.93 (0.83, 1.05)	Ref	1.05 (0.87, 1.26)
	OCD	Ref	1.00 (0.77, 1.31)	Ref	1.20 (0.81, 1.77)
	Non-OCD	Ref	0.92 (0.82, 1.05)	Ref	0.99 (0.81, 1.21)
	Mood disorder	Ref	0.93 (0.80, 1.07)	Ref	1.19 (0.95, 1.49)
	Depression	Ref	0.92 (0.80, 1.08)	Ref	1.21 (0.96, 1.52)
	Severe depression	Ref	0.92 (0.78, 1.08)	Ref	1.18 (0.92, 1.51)
	Suicide	Ref	0.96 (0.66, 1.41)	Ref	0.86 (0.45, 1.66)
	Antidepressant drug use	Ref	0.99 (0.90, 1.09)	Ref	1.12 (0.96, 1.31)

ART is assisted reproductive techniques
CI is confidence interval (95%)
IVF is in-vitro fertilization
ICSI is intracytoplasmic sperm injection
OCD is obsessive compulsive disorder

a. The model was adjusted for birth year, parents' age at delivery, origin, highest education, parity, civil status, region of residence, maternal smoking status, maternal overweight and obesity, maternal medical conditions, and parental psychiatric history.

© 2021 Wang C et al. *JAMA Psychiatry.*

eTable 6. Association Between ART and Different Antidepressant Use in Individuals After Multiple Imputation or Complete Information

	Antidepressants	Number of Events (exposed/non-exposed)	Crude hazard ratio[a] (lower CI, upper CI)	Adjusted hazard ratio[b] (lower CI, upper CI)
**Multiple imputation**				
ART compared to all other				
	SSRIs	2165/98149	1.03 (0.99, 1.07)	1.04 (1.00, 1.09)
	Non-SSRIs	727/37721	0.98 (0.91, 1.05)	1.07 (0.99, 1.15)
ART compared to non-ART with parental infertility				
	SSRIs	2165/7799	0.96 (0.92, 1.01)	0.99 (0.94, 1.04)
	Non-SSRIs	727/2901	**0.90 (0.83, 0.98)**	1.00 (0.92, 1.09)
ICSI compared to standard IVF				
	SSRIs	710/1455	1.02 (0.93, 1.12)	1.04 (0.95, 1.14)
	Non-SSRIs	225/502	1.07 (0.91, 1.26)	1.08 (0.92, 1.27)
Frozen compared to fresh embryo transfer				
	SSRIs	208/1594	1.09 (0.94, 1.26)	1.12 (0.97, 1.30)
	Non-SSRIs	76/524	1.25 (0.98, 1.59)	1.27 (0.99, 1.62)
**Complete-case analysis**				
ART compared to all other				
	SSRIs	1696/77199	0.98 (0.94, 1.03)	1.03 (0.98, 1.08)
	Non-SSRIs	564/29163	0.94 (0.87, 1.03)	1.05 (0.97, 1.14)
ART compared to non-ART with parental infertility				
	SSRIs	1696/6968	**0.93 (0.89, 0.99)**	0.99 (0.93, 1.04)
	Non-SSRIs	564/2567	**0.89 (0.81, 0.97)**	0.98 (0.89, 1.08)
ICSI compared to standard IVF				
	SSRIs	574/1122	0.96 (0.86, 1.06)	0.97 (0.87, 1.08)
	Non-SSRIs	170/394	0.97 (0.80, 1.17)	0.96 (0.80, 1.17)
Frozen compared to fresh embryo transfer				
	SSRIs	168/1272	1.05 (0.89, 1.23)	1.08 (0.92, 1.27)
	Non-SSRIs	59/408	1.22 (0.92, 1.60)	1.23 (0.93, 1.62)

ART is assisted reproductive techniques
CI is confidence interval (95%)
IVF is in-vitro fertilization
ICSI is intracytoplasmatic sperm injection
SSRI is selective serotonin receptor inhibitor

a. The model was stratified Cox proportional hazard regression was stratified by birth year.
b. The model was also adjusted for parents' age at delivery, origin, highest education, parity, civil status, region of residence, maternal smoking status, maternal overweight and obesity, maternal medical conditions, and parental psychiatric history.

© 2021 Wang C et al. *JAMA Psychiatry.*

eTable 7. Association Between ART Specific Procedures and Different Antidepressant Use in Individuals After Multiple Imputation or Complete Information

		Adjusted hazard ratio[a] (lower CI, upper CI)			
		**Type of fertilization**		**Type of embryo transfer**	
		**IVF**	**ICSI**	**Fresh**	**Frozen**
**Multiple imputation**					
ART specific procedures compared to non-ART with parental infertility					
	SSRIs	0.97 (0.91, 1.03)	1.04 (0.96, 1.13)	0.97 (0.92, 1.03)	1.06 (0.92, 1.22)
	Non-SSRIs	0.97 (0.88, 1.07)	1.05 (0.91, 1.21)	0.94 (0.85, 1.04)	1.18 (0.94, 1.49)
ART specific procedures directly compared					
	SSRIs	Ref	1.04 (0.95, 1.14)	Ref	1.12 (0.97, 1.30)
	Non-SSRIs	Ref	1.08 (0.92, 1.27)	Ref	1.27 (0.99, 1.62)
**Complete-case analysis**					
ART specific procedures compared to non-ART with parental infertility					
	SSRIs	0.99 (0.92, 1.05)	0.98 (0.89, 1.07)	0.98 (0.92, 1.04)	1.02 (0.88, 1.19)
	Non-SSRIs	1.00 (0.89, 1.11)	0.93 (0.79, 1.10)	0.93 (0.83, 1.04)	1.15 (0.89, 1.49)
ART specific procedures directly compared					
	SSRIs	Ref	0.97 (0.87, 1.08)	Ref	1.08 (0.92, 1.27)
	Non-SSRIs	Ref	0.96 (0.80, 1.17)	Ref	1.23 (0.93, 1.62)

ART is assisted reproductive techniques
CI is confidence interval (95%)
IVF is in-vitro fertilization
ICSI is intracytoplasmic sperm injection
SSRI is selective serotonin receptor inhibitor

a. The model was adjusted for birth year, parents' age at delivery, origin, highest education, parity, civil status, region of residence, maternal smoking status, maternal overweight and obesity, maternal medical conditions, and parental psychiatric history.

© 2021 Wang C et al. *JAMA Psychiatry.*
